# fNIRS can robustly measure brain activity during memory encoding and retrieval in healthy subjects

**DOI:** 10.1038/s41598-017-09868-w

**Published:** 2017-08-25

**Authors:** Sahar Jahani, Antoniu L. Fantana, David Harper, James M. Ellison, David A. Boas, Brent P. Forester, Meryem A. Yücel

**Affiliations:** 1 MGH/HST Athinoula A. Martinos Center for Biomedical Imaging, Department of Radiology, Massachusetts General Hospital, Harvard Medical School, Charlestown, MA USA; 2 Control and Intelligent Processing Center of Excellence, School of Electrical and Computer Engineering, College of Engineering, University of Tehran, Tehran, Iran; 3 Department of Neurology, Brown University, RI Providence, USA; 4 Division of Geriatric Psychiatry at McLean Hospital, Belmont, MA USA; 5 Swank Memory Care Center, Christiana Care Health System, DE Wilmington, USA

## Abstract

Early intervention in Alzheimer’s Disease (AD) requires novel biomarkers that can capture changes in brain activity at an early stage. Current AD biomarkers are expensive and/or invasive and therefore unsuitable for use as screening tools, but a non-invasive, inexpensive, easily accessible screening method could be useful in both clinical and research settings. Prior studies suggest that especially paired-associate learning tasks may be useful in detecting the earliest memory impairment in AD. Here, we investigated the utility of functional Near Infrared Spectroscopy in measuring brain activity from prefrontal, parietal and temporal cortices of healthy adults (n = 19) during memory encoding and retrieval under a face-name paired-associate learning task. Our findings demonstrate that encoding of novel face-name pairs compared to baseline as well as compared to repeated face-name pairs resulted in significant activation in left dorsolateral prefrontal cortex while recalling resulted in activation in dorsolateral prefrontal cortex bilaterally. Moreover, brain response to recalling was significantly higher than encoding in medial, superior and middle frontal cortices for novel faces. Overall, this study shows that fNIRS can reliably measure cortical brain activation during a face-name paired-associate learning task. Future work will include similar measurements in populations with progressing memory deficits.

## Introduction

Early detection of Alzheimer’s disease (AD) is critical to facilitate timely intervention to attenuate or stop the progressive cognitive decline. Loss of memory for recently experienced events or the so-called “episodic memory” is the first symptom of amnesia in patients with AD and thus a large number of studies have been conducted to investigate the underlying brain phsyiology^1–4^. Normal aging also affects episodic memory capability^2, 5–7^ as well as the ability to recall the spatiotemporal details of an event^8, 9^, necessitating a method for differentiating between normal and pathological memory decline. Such a method is critical as it has the potential to capture the functional abnormalities before any observable structural change.

Remembering proper names, the paired-associate learning task (PAL), which requires the use of episodic memory is the most common problem among elderly^2, 10–15^. Several studies suggested that the PAL task could be useful in early detection of memory impairment^16, 17^. In the PAL task, one item of a pair is used to cue recall of the other item, and the subject’s ability to form an association between the paired items is assessed^15^. While each item in the task may come from different domains, a formative association between domains is attempted during learning. The PAL task is widely used for assessing memory performance in the young and elderly population as well as patients with Mild Cognitive Impairment (MCI) and Alzheimer’s Disease^2, 11–13, 18–20^. Specific regions of the hippocampus and prefrontal cortices have been identified as critical to the success of memory encoding in both young and healthy elderly subjects during a face-name PAL task^2, 18^. In particular, the dorsolateral and ventrolateral prefrontal cortex (DLPFC and VLPFC) have been shown to be associated with the encoding and recalling phase of face-name PAL task^20^. Several fMRI studies have shown that left inferior frontal^21, 22^ and superior temporal cortices^15, 23^ are activated during encoding of novel faces, while frontal and parietal cortices are activated during the encoding of the same faces^15^. Increasing task difficulty during the paired-associate learning task results in enhanced involvement of the same, rather than an additional, network of brain regions in both healthy controls and patients with AD^12^. In addition, in AD patients, greater recruitment of the same brain regions was observed, presumably as a means of compensating for cognitive impairment^11^.

Importantly, even if there is no overt difference in memory performance between healthy elderly and early stages of AD, functional neuroimaging methods can detect changes in brain physiology at earlier stages. Unfortunately, inexpensive and practical screening tests currently used are not sensitive or specific to the actual changes in brain physiology at very early stages. Positron emission tomography (PET) amyloid imaging and cerebrospinal fluid protein analysis are optimal biomarkers for detecting underlying AD neuropathology but are expensive and invasive^24–26^. Functional near-infrared spectroscopy (fNIRS) is a relatively new optical imaging technology that uses light in the near-infrared spectrum to noninvasively monitor the hemodynamic responses evoked by neural activity through measuring the changes in oxy and deoxy-hemoglobin concentrations in the cerebral cortex^27^. The increased blood supply to the area of neural activation typically results in an increase in oxy-hemoglobin concentration (HbO) while a decrease is observed in deoxy-hemoglobin concentration (HbR) due to the blood’s wash-out effect. The HbO and HbR responses from fNIRS measurements have been shown to be spatially and temporally correlated with the blood oxygen level-dependent signal (BOLD) obtained by fMRI^28–30^. The advantage of fNIRS over other imaging modalities is that it is inexpensive, non-invasive and portable and thus a great candidate as a screening tool for early detection of persons at elevated risk for AD.

fNIRS has been recently employed to investigate various aspects of memory such as encoding and retrieval of story^31^, episodic encoding and retrieval of taste^32^ and the effect of music on verbal memory encoding^33^. However, to our knowledge, brain activation in prefrontal cortex during paired associate learning has been investigated only by one research group using word pairs^19^. In the present study, we used an fNIRS system for monitoring a wider brain area i.e. the prefrontal, parietal and temporal cortices of healthy subjects during a face-name paired-associate learning task previously implemented in an fMRI study^14^. Moreover, we have included additional short separation detectors to separate the brain activity and the global physiological changes in scalp, and digitized 3D locations of optodes which allowed easy comparison with fMRI literature. Our findings were consistent with prior fMRI and PET literature and proved that fNIRS can robustly measure brain activity during a face-name paired associate learning task. The future work will include measurements on healthy elderly and patients with memory deficits such as patients with MCI or AD to differentiate the brain responses of different populations. If characteristic alterations of hemodynamic responses to encoding and retrieval can be linked with disease, fNIRS may offer a surrogate biomarker for early detection and assessment of memory impairment.

## Results

**Figure 1:**
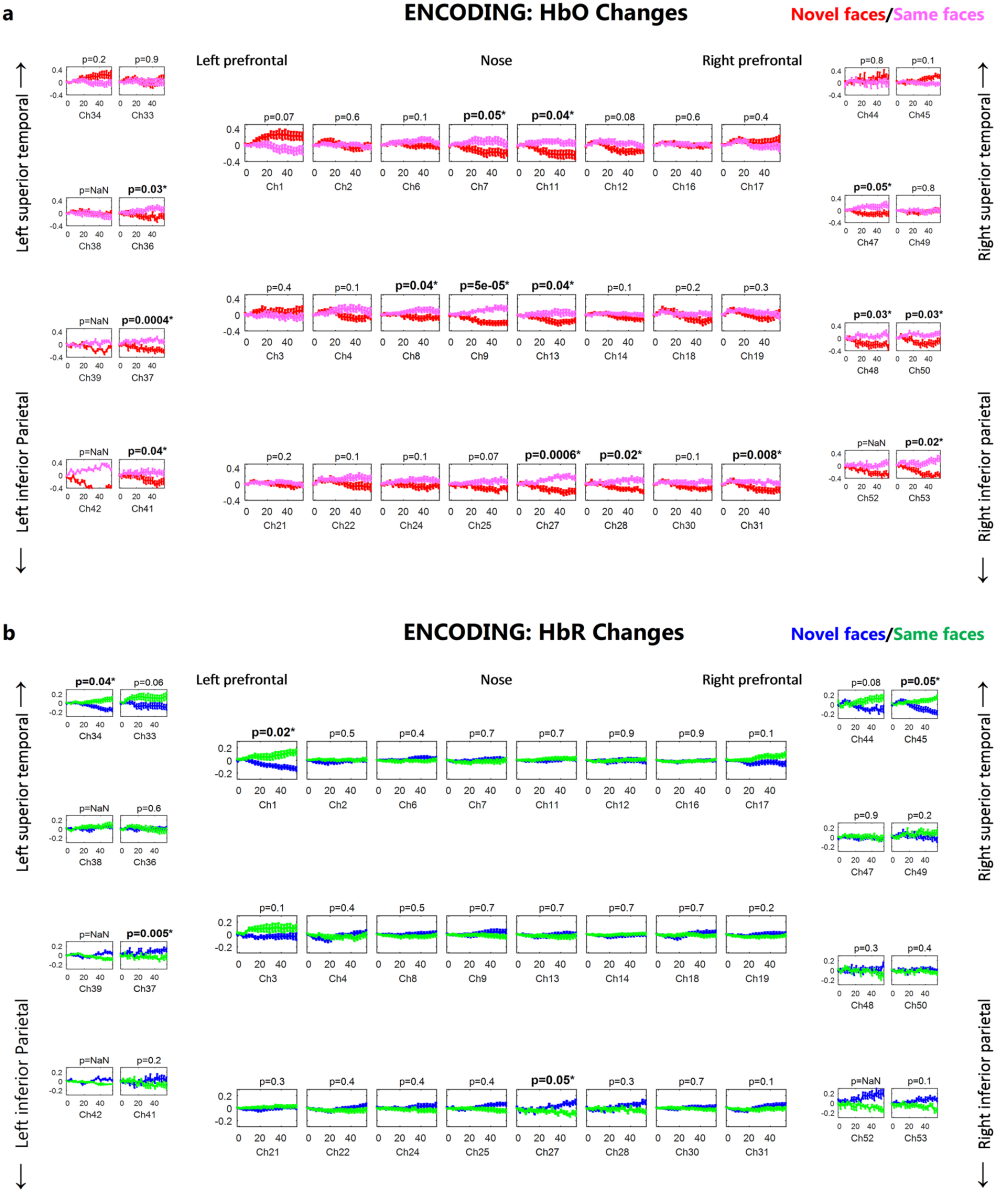
Group results for encoding of same faces vs. novel faces (**a**) HbO changes, (**b**) HbR changes in µM. The p-value for the differences between novel and same faces for each channel is written on the top of each channel. The p-values shown are corrected for multiple comparisons (alpha = 0.05). The HbO/HbR changes are plotted in red/blue for novel faces and pink/green for same faces for the duration of −2 to 55 seconds. The stimulus was presented from 0 to ~50 seconds. The standard error of the response across the group is indicated. The channels where more than 5 subjects are removed due to poor signal quality are not included in statistical analysis and hence no p-value is provided (NaN).

**Figure 2:**
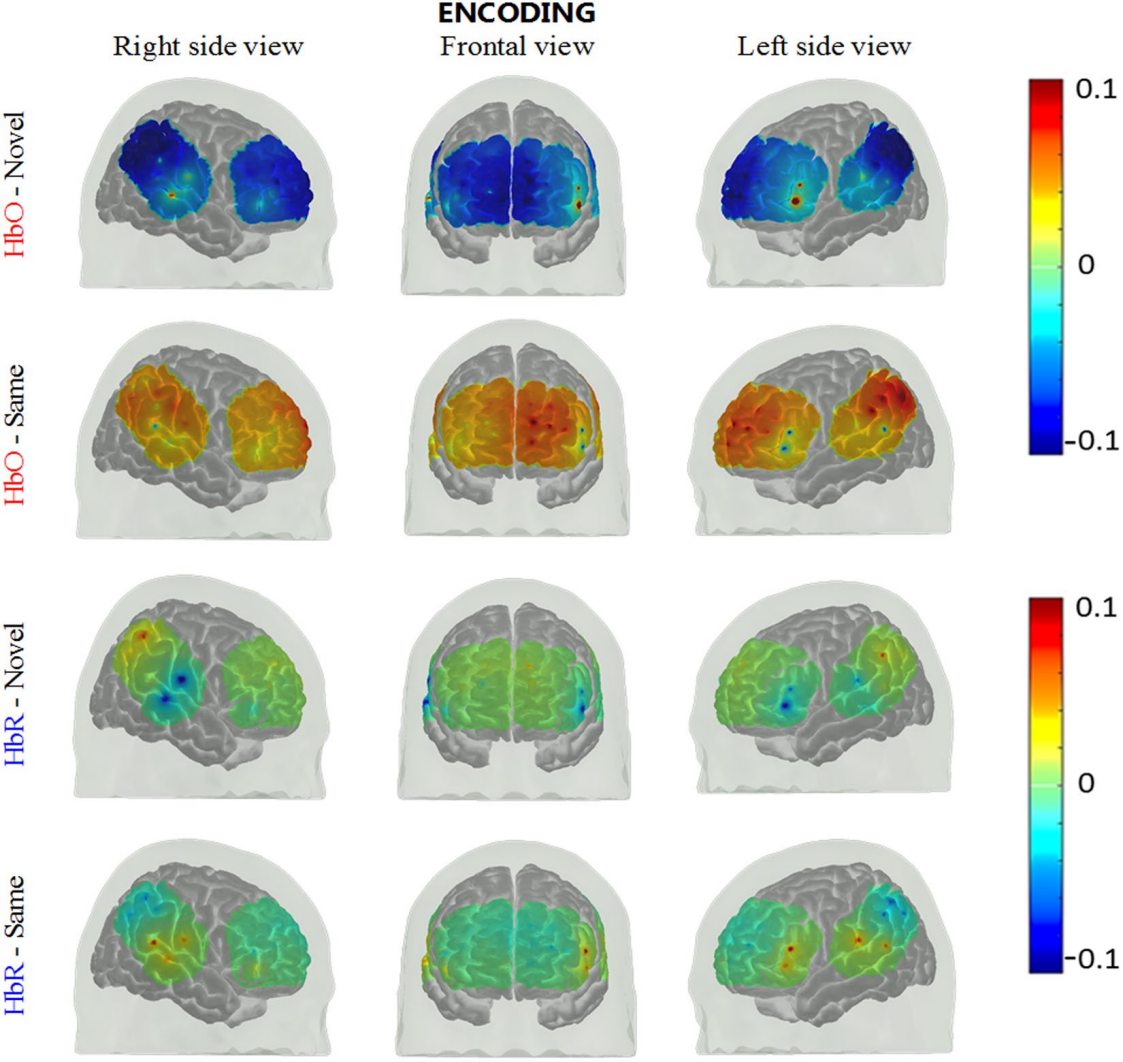
Group mean HbO and HbR hemodynamic responses overlayed over the brain surface for encoding of novel and same faces. The displayed concentration changed was averaged from t = 25 to 50 sec. The color bar indicates the scale of the concentration change in µM units.

The group mean (n = 19 subjects) temporal traces of the oxy-hemoglobin and deoxy-hemoglobin concentration changes from baseline levels during encoding of novel and same faces are depicted in Fig. 1. The group mean spatial results averaged over t = 25 to 50 seconds are also overlayed on brain surface for better visual presentation of the different brain regions involved in encoding of novel and same faces (Fig. 2).

### Encoding of novel faces

There was a significant increase in the HbO concentration from baseline levels in left inferior frontal cortex (ch 1, paired t-test, FDR corrected p-value < 0.05) and a significant decrease in lateral superior medial frontal, right superior frontal, lateral inferior parietal cortices (ch 7, 9, 11, 12, 13, 25, 27, 28, 30, 37, 48, 52 and 53, paired t-test, FDR corrected p-value < 0.05) (See methods for the location of each channel). On the other hand, the HbR concentration significantly decreased in left inferior frontal and left superior temporal cortex (ch 1 and 34, paired t-test, FDR corrected p-value < 0.05). The number of channels used for each subject, MNI coordinates of the channels that show significant differences between novel and same faces, their corresponding Brodmann areas along with their p-values are shown in Fig. 8.

### Encoding of previously presented (“same”) faces

HbO showed a significant increase from baseline in lateral medial superior frontal and left inferior parietal cortices (ch 9, 27 and 37, paired t-test, FDR corrected p-value < 0.05) and HbR showed a significant decrease from baseline in right inferior parietal cortex (ch 52, paired t-test, FDR corrected p-value < 0.05). Right inferior frontal cortex showed significant increases in HbR (ch 17, paired t-test, FDR corrected p-value < 0.05).

### Encoding of novel faces vs same faces

Only one channel showed marginally significant difference in HbO between the encoding of novel and same faces where HbO increase was higher for the novel faces (ch 1; paired t-test, FDR corrected p-value = 0.07) (Fig. 1). The encoding of same faces resulted in higher HbO concentration compared to novel faces in the rest of the channels that show significant differences between the two (ch 7, 8, 9, 11, 13, 27, 28, 31, 36, 37, 41, 47, 48, 50 and 53; paired t-test, FDR corrected p-value < 0.05). The right medial superior frontal, left inferior parietal regions (ch 27 and 37, paired t-test, FDR corrected p-value < 0.05) showed significantly higher HbR response for novel faces while left inferior frontal, lateral superior temporal areas showed lower HbR compared to same faces (ch 1, 34 and 45, paired t-test, FDR corrected p-value < 0.05).

### Inter-subject variability during Encoding (novel vs. same faces): Medial and Superior Frontal Cortex

**Figure 3:**
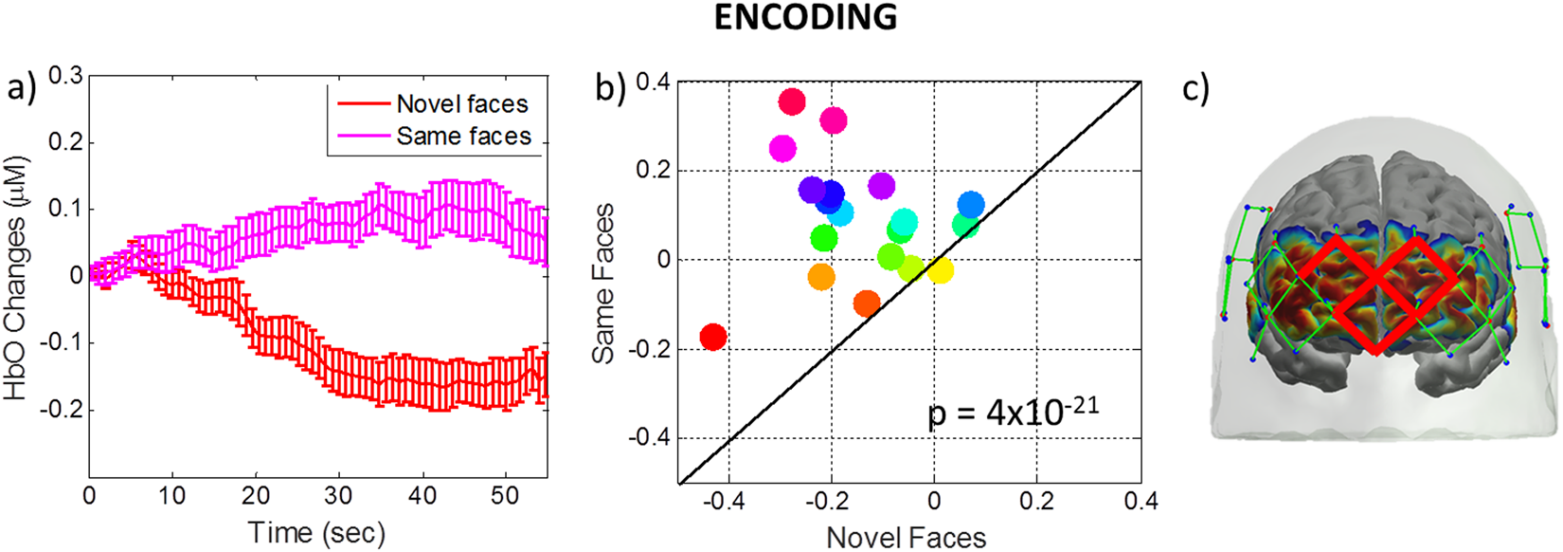
HbO changes in medial and superior frontal cortex averaged across all subjects during the novel and same face-name pair blocks. HbO changes (µM) during encoding of novel (red) and same (pink) faces (3a) averaged over the ROI defined by the channels marked in red on the head (3c). The scatter plot (3b) shows the response averaged over 25 to 50 sec for each individual subject. Each subject is depicted as a color-filled circle. The results indicate that the HbO responses are higher for each subject for Same Faces than for Novel Faces for all but one subject. The p-value is for the difference between novel and same faces over 25 to 50 sec.

We further investigated the inter-subject variability in brain regions where localized brain activity differs with statistical significance between the encoding of novel versus same faces. As seen from Figs 1 and 2, there is a localized HbO decrease/increase in medial and superior frontal cortex for novel/same faces respectively. The hemodynamic response in the channels in this region is averaged to obtain Fig. 3. This specific ROI showed the most significant difference between two conditions (p-value = 4 × 10^−21^). The scatter plot shows the response for each individual subject averaged over the time period 25 to 50 sec and reveals that the HbO responses are higher for each subject for same faces than for novel faces for all but one subject.

### Recalling of novel faces

**Figure 4:**
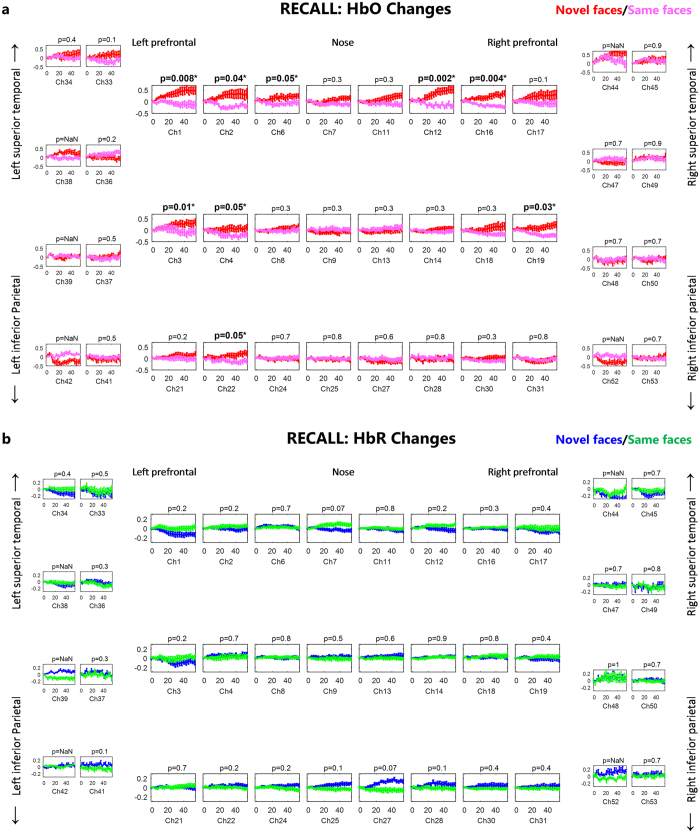
Group results for recalling of same faces vs. novel faces (**a**) HbO changes, (**b**) HbR changes in µM. The p-value for the differences between novel and same faces for each channel is written on the top of each channel. The p-values shown are corrected for multiple comparisons (alpha = 0.05). The HbO/HbR changes are plotted in red/blue for novel faces and pink/green for same faces for the duration of −2 to 55 seconds. The stimulus was presented from 0 to ~50 seconds. The standard error of the response across the group is indicated. The channels where more than 5 subjects are removed due to poor signal quality are not included in statistical analysis and hence no p-value is provided (NaN).

**Figure 5:**
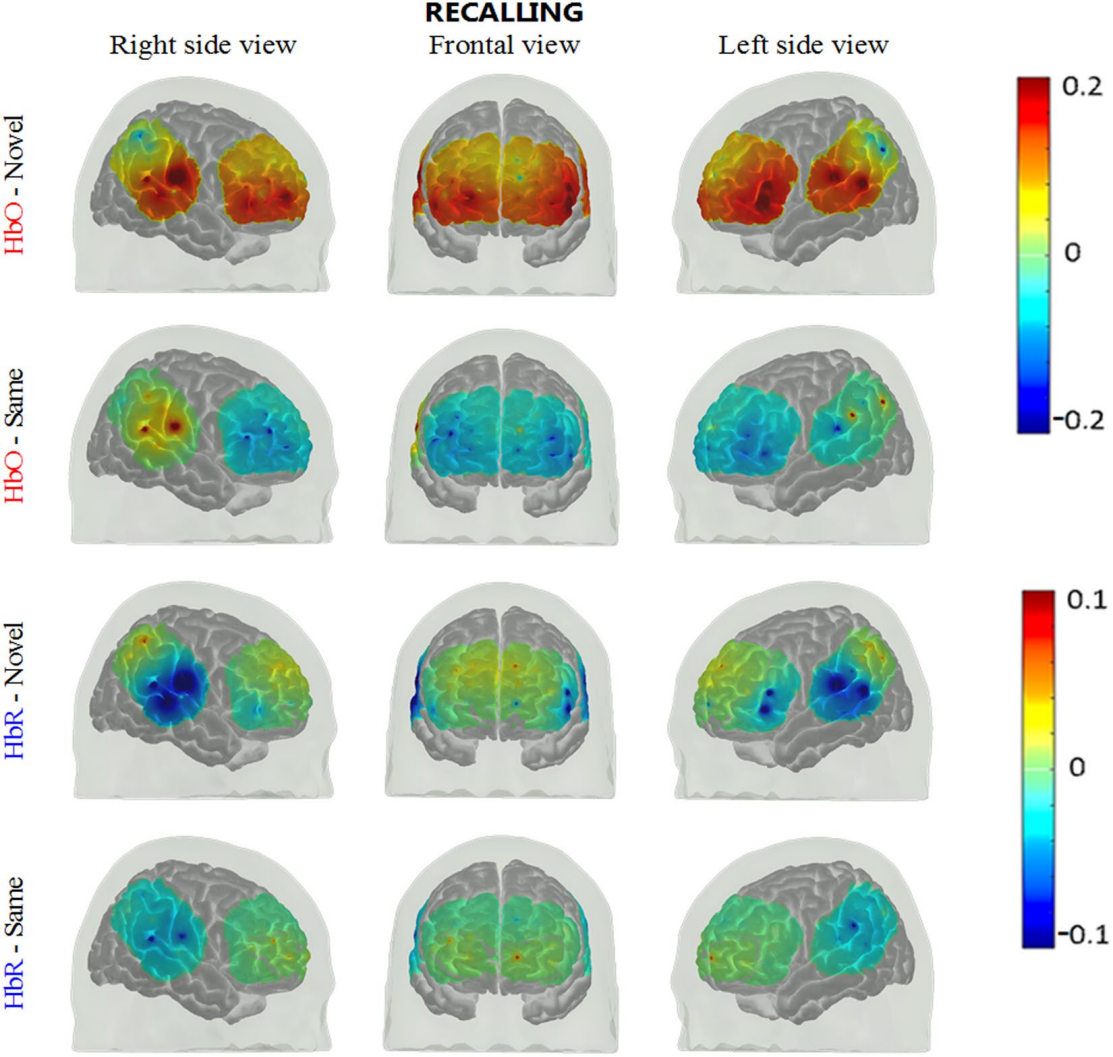
Group mean HbO and HbR hemodynamic responses overlayed over brain surface for recalling of novel and same faces. The displayed concentration changed was averaged from t = 25 to 50 sec. The color bar indicates the scale of the concentration change in µM units.

The group mean oxy-hemoglobin and deoxy-hemoglobin concentration changes from n = 19 subjects during recalling of novel and same faces are shown in Fig. 4. The mean HbO and HbR during t = 25 to 50 sec are overlayed on brain surface in Fig. 5. HbO increased from baseline in left inferior frontal, right superior frontal and right middle frontal (ch 1, 3, 12 and 16, paired t-test, FDR corrected p-value < 0.05), while HbR showed decreases from baseline in left inferior frontal, right superior temporal cortices (ch 1, 3, 45 and 49, paired t-test, FDR corrected p-value < 0.05) and showed increases from baseline in right superior medial cortex (ch 27, paired t-test, corrected p-value < 0.05).

### Recalling of same faces

During the recalling of same faces, no channel showed a significant increase in HbO while many channels showed a significant decrease in HbO, including lateral middle frontal, right superior frontal, and right inferior frontal cortices (ch 2, 4, 16 and 19, paired t-test, FDR corrected p-value < 0.05). For HbR, there was not any significant decrease or increase from the baseline in recalling of the same faces.

### Recalling of novel faces vs same faces

We observed a significant novel versus same face contrast in a wider area of frontal cortex during the recalling phase in comparison to the encoding phase (Compare Figs 1a and 4a). The lateral inferior frontal, right superior frontal and lateral middle frontal cortices (ch 1, 2, 3, 4, 6, 12, 16, 19 and 22, paired t-test, FDR corrected p-value < 0.05) showed higher HbO for novel faces than same faces (Fig. 4). Right medial superior frontal cortices showed higher HbR for recalling of the novel faces than same faces (ch 27, paired t-test, FDR corrected p-value = 0.07, marginal significance) while higher HbR for recalling of same faces was observed in left medial superior frontal cortex (ch 7, paired t-test, FDR corrected p-value = 0.07, marginal significance).

### Inter-subject variability during Recalling (novel vs. same faces): Inferior to Middle Frontal Cortex

**Figure 6:**
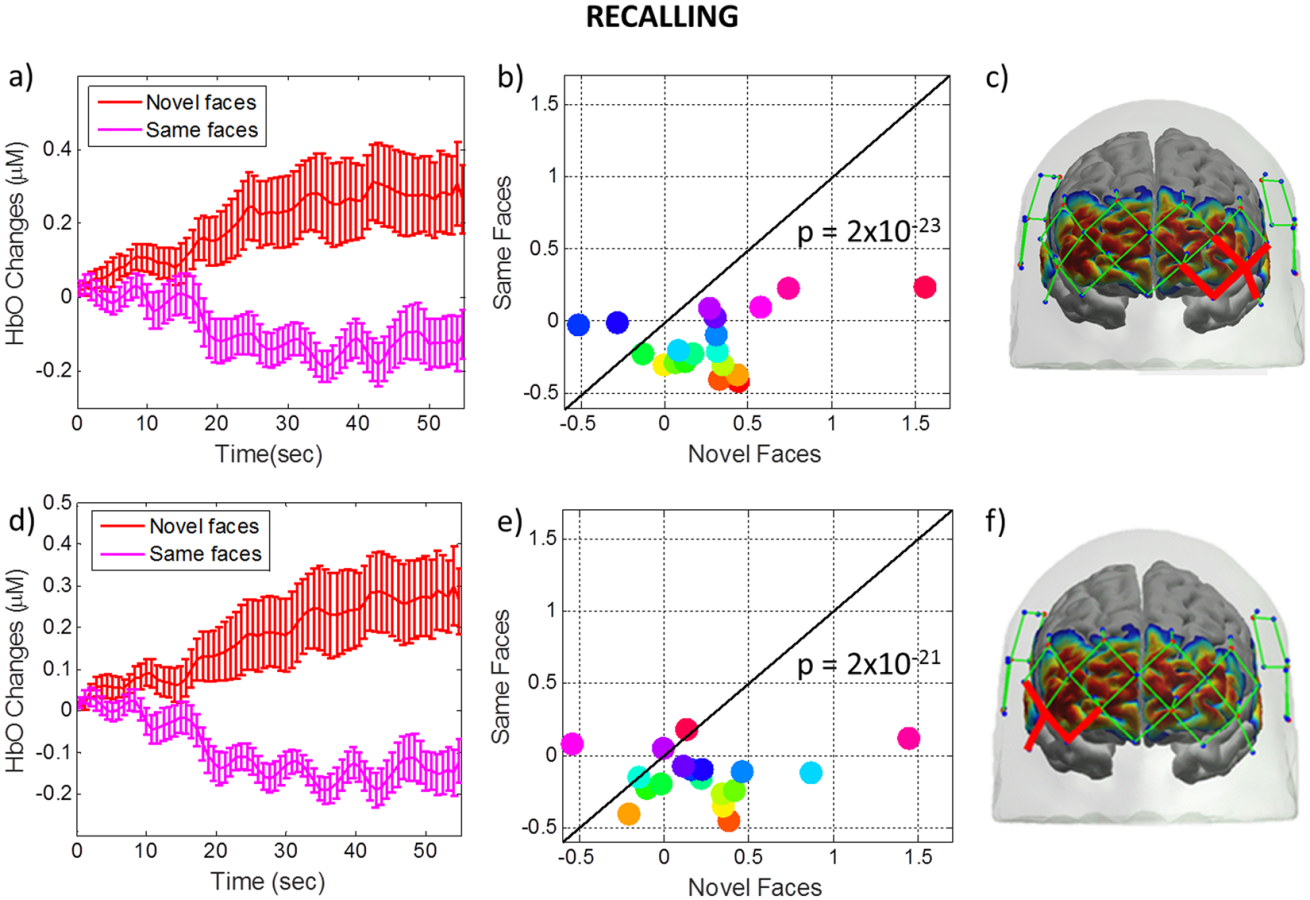
HbO changes in inferior to middle frontal cortex averaged across all subjects during the novel and same face-name pair blocks. HbO changes (µM) during recalling of novel (red) and same (pink) faces (6a and 6d) averaged over the ROI defined by the channels marked in red on the head (6c and 6f). The scatter plot (6b and 6e) shows the response averaged over 25 to 50 sec for each individual subject. Each subject is depicted as a color-filled circle. The results indicate that the HbO responses are higher for each subject for Novel Faces than for Same Faces for all but 2 to 4 subjects. The p-value is for the difference between novel and same faces over 25 to 50 sec.

During recalling, we observed a bilateral HbO response in inferior and middle frontal cortex (See Figs 4 and 5). We have defined our ROI as the combination of channels that showed statistically significant difference between two conditions in this region (Fig. 6, red channels on the head). The mean response over the channels in these regions is shown in Fig. 6. During recall, the HbO response to novel faces was significantly higher than the response to same faces in both ROIs (left: p-value = 3 × 10^−20^, right: p-value = 2 × 10^−23^). The scatter plot shows the response for each individual subject averaged over the time period 25 to 50 sec.

### Recalling vs. Encoding of novel and same face-name pairs

**Figure 7:**
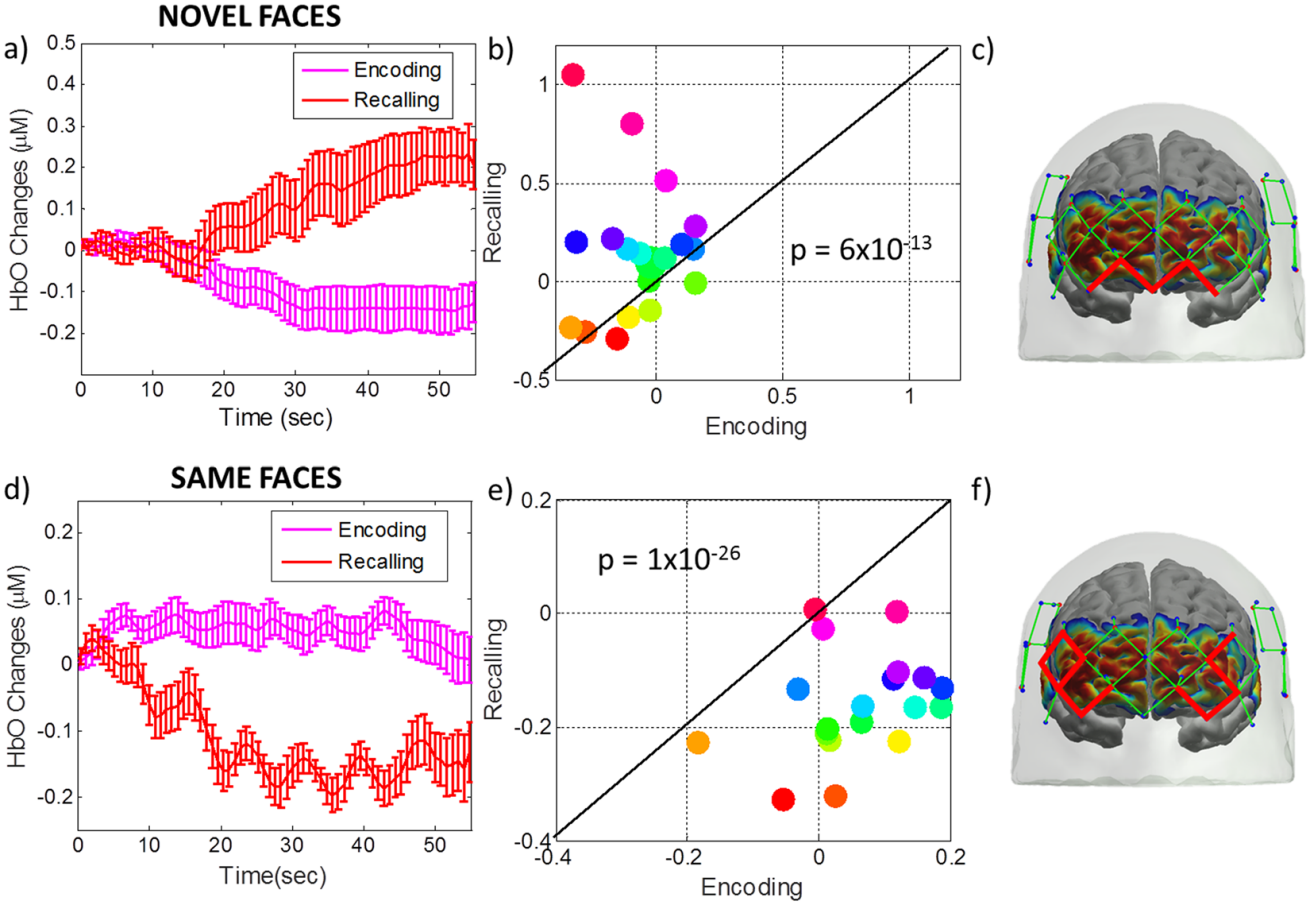
Group average HbO changes (µM) during recalling (red) and encoding (pink) averaged over (**a**) medial and superior frontal cortices for novel faces and (**d**) lateral middle, lateral superior and left inferior frontal cortices for same faces. HbO changes (µM) averaged over the ROI defined by the channels marked in red on the head (**c** and **f**). The scatter plot (**b** and **e**) shows the response averaged over 25 to 50 sec for each individual subject. Each subject is depicted as a different color-filled circle. The p-values are for the difference between recalling vs. encoding of (**b**) novel and (**e**) same faces over 25 to 50 sec.

We observed significant differences between recalling and encoding of novel as well as same faces in the frontal cortex. Recalling of novel faces produced a significantly higher response in superior and middle frontal cortex compared to encoding (Fig. 7c, red channels on the head) (p-value = 6 × 10^−13^). The average HbO time course over the ROI defined in Fig. 7c is displayed in Fig. 7a. The scatter plot shows the response for each individual subject averaged over the time period 25 to 50 sec (Fig. 7b). In contrast, recalling of same faces resulted in lower hemodynamic responses in middle, superior and inferior frontal cortex compared to encoding. (Fig. 7d and f), particularly in the middle frontal cortex (p-value = 1 × 10^−26^). The results were consistent across subjects (Fig. 7e).

**Figure 8:**
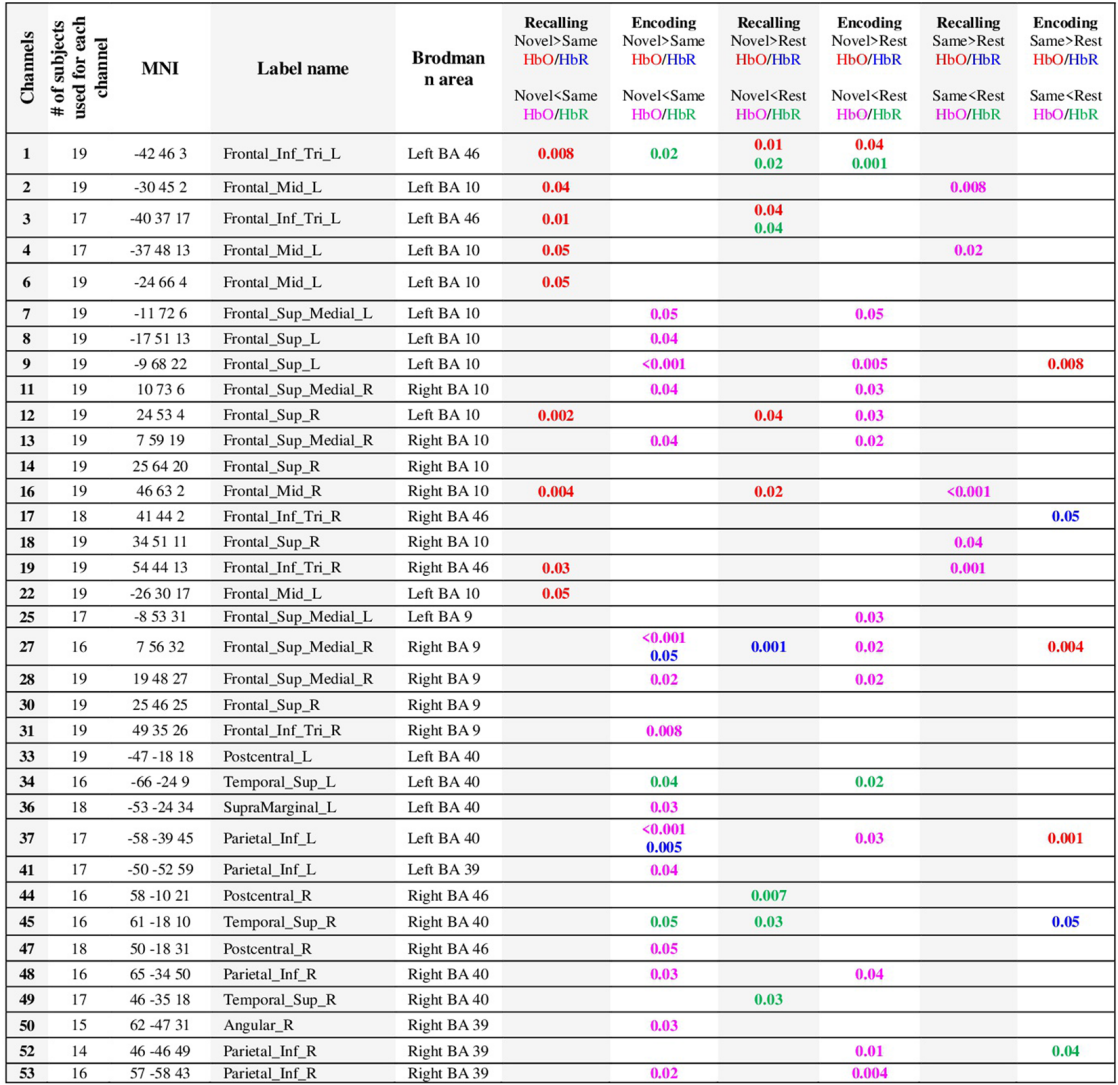
Number of subjects used for each channel, MNI coordinates and brain regions of the channels that showed statistically significant changes from resting or between conditions, and their corresponding p-values in comparing encoding and recalling of novel vs. same faces.The p-values shown are corrected for multiple comparisons. Brodmann areas for each MNI coordinate are obtained from http://sprout022.sprout.yale.edu/mni2tal/mni2tal.html.

### Memory performance and brain activation during retrieval of novel faces

**Figure 9:**
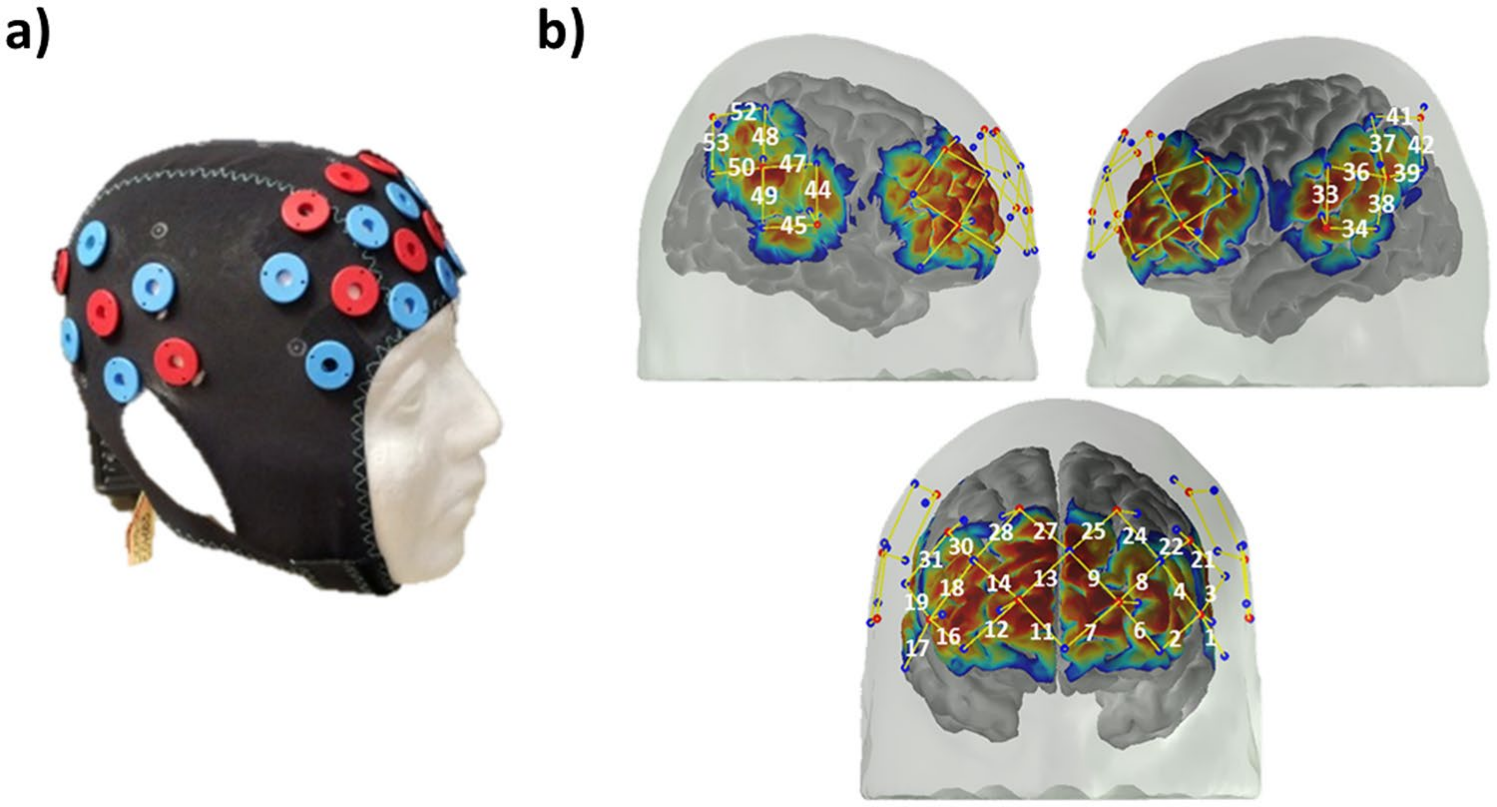
The designed probe, (**a**) Location of sources (red circles) and detectors (blue circles) on the cap, (**b**) Sensitivity profile for the probe shown on the left. The yellow numeration corresponds to the channel number in figures and tables. The color scale spans the sensitivity logarithmically from 0.01 (blue) to 1 (red). (Short separation channel numbers are omitted in this figure).

We further investigated the relationship between brain activation and accuracy of recall by calculating the Pearson correlation between accuracy of the recalling of the novel face-name pairs and the mean of HbO and HbR change in time interval of 25 to 50 seconds during the recalling blocks. Better accuracy performance was negatively correlated with an HbO increase in inferior frontal cortex (channels with correlation coefficients in parenthesis: ch1 (−0.22), ch19 (−0.27) and ch21 (−0.27), middle frontal cortex (ch2 (−0.33), ch16 (−0.23), ch18 (−0.43)), superior frontal cortex (ch12 (−0.22), ch30 (−0.26)) and superior temporal cortex (ch34 (−0.24)) (p-value < 0.05). Please refer to Fig. 4a or 9 for the location of channels.

## Discussion

Episodic memory is the capacity to remember individual features of an event that allow one experience to be separated from another and depends on the ability to encode associations between the different components that constitute the event^34, 35^. Associations can be formed either between the items that are the primary focus of attention such as words or pictures, or between items and the context, i.e. the background information^36^. PET and fMRI studies have demonstrated that episodic memory is supported by the involvement of prefrontal cortex^37–39^ and temporal lobe^15, 23, 40, 41^. Particularly, activation in dorsolateral prefrontal cortex (DLPFC)^42^, ventrolateral prefrontal cortex (VLPFC)^36, 43–45^, temporal lobe^15, 23, 36, 40^, and left inferior PFC^21, 22, 46, 47^ are associated with encoding and left DLPFC^48, 49^, VLPFC^31, 50^, left inferior PFC^51^, and inferior temporal^49^ are associated with recalling. There are some inconsistencies in literature in terms of which sub-regions are involved. Involvement of different sub-regions in different studies may reflect investigation of different types of associations involved in the memory tasks^36^.

In this study, encoding of novel faces resulted in significant activation in left inferior frontal cortex and deactivation of medial to superior frontal and lateral inferior parietal cortices. Several fMRI and PET studies on memory encoding reported activation of left inferior frontal gyrus during encoding of words or pictures^22, 46, 47, 52–57^. Moreover, an fMRI study has shown that the greater involvement of this region during the memory encoding phase increases the probability of successful retrieval^58^. These results point to the key role of the left inferior frontal cortex in memory formation. Engagement of superior temporal cortex has also been observed during encoding the information of sound categories^40^, encoding of novel faces^15^, encoding of speech sequence probabilities^41^ and face perception^23^. Although our results show HbO increases from baseline in the right temporal cortex during encoding of novel faces, the statistical significance did not survive after FDR correction.

The regions deactivated during encoding of novel faces: medial frontal and inferior parietal cortices, showed activation during encoding of same faces. Interestingly, these regions overlap with the default mode network (DMN)^59^. DMN, which is also referred to as the task negative network^60–62^, is known to be activated during passive states such as rest, daydreaming or when a subject is not involved in a cognitively demanding task^59^. As subjects were shown the same faces before the experiment and the fact that the same faces were repeated many times during the two encoding runs, the repetition of same faces during encoding did not require subjects to perform any task and the brain was at a wakeful rest which may result in activation of the DMN. Another possible explanation for the activation during the presentation of the same faces could be the face recognition process. An fMRI study revealed activation in the right medial frontal gyrus, the right anterior cingulate gyrus, and parietal association cortices during recognition of previously presented words^56^. This and other fMRI studies concluded that the activation of frontal and parietal cortices may be due to the recognition of previously experienced stimuli^15, 55, 63^. On the other hand, several fMRI studies observed strong deactivation in the medial frontal cortex which suggests that memory encoding of novel faces requires an increase in attention and thus may result in the suppression of DMN during the encoding phase^64–70^. In our study, recalling of novel faces activated lateral inferior and middle frontal cortices and right superior frontal cortex. However, we did not observe significant activation in the medial frontal cortex or elsewhere during the recalling of same faces. It may be that even recalling of same faces requires a certain level of attention and hence DMN was not activated.

Recalling of novel face-name pairs produced significantly higher HbO increase in superior and middle frontal cortex compared to encoding. Moreover, the brain response during the recalling phase was spatially broader than the brain response during encoding of novel face-name pairs. This is in accordance with previous fNIRS and fMRI studies that showed higher and broader activation during the recalling phase in the frontal cortex^19, 31, 49, 71–74^. For instance, Mandzia and colleagues found higher brain activation in prefrontal regions and the mediotemporal lobe during recalling in comparison to encoding^72^. In their study, they suggest that the greater involvement of neural activity during recalling may be due to a need for greater cognitive resources such as involvement of attention or semantic processing. As a matter of fact, harder tasks are known to require both higher brain activation and involvement of broader brain areas when compared to easy tasks^12^.

Improved accuracy in recalling the face-name pairs was negatively correlated with HbO in lateral inferior frontal and middle and superior frontal regions. It is encouraging that most of the channels were among the ones which also showed significant activation from baseline during recalling. The correlation was relatively low, which could be explained by the relatively low sample size of our study. Consistent with our results, an fMRI study also suggests a negative correlation between performance and brain activation in dorsolateral prefrontal cortex in healthy subjects^75^; however, their result was not statistically significant. The correlation may indicate that better-performing subjects have higher baseline activity in these regions and need not increase from baseline levels as much as the poor performing subjects who put more effort to recall and thus increase the brain activation more.

One limitation of fNIRS is its penetration depth which allows detection of brain signals only from the cerebral cortex. Brain areas such as hippocampus, for instance, which is intensely studied in the fMRI literature regarding its role in AD, cannot be detected by fNIRS. However, prior literature showed evidence of the association of cortical regions with the success of memory encoding and retrieval in addition to the hippocampus, such as DLPFC and VLPFC, temporal and parietal cortices^2, 18, 20^ which are detectable by fNIRS. This gives us confidence that it is possible to detect hemodynamic changes related to memory encoding and retrieval in cortex using fNIRS technology.

Cognitive activity loads may uncover subtle brain functional abnormalities not present during baseline cognitive states. While less demanding tasks result in similar activation levels during health and disease, more demanding tasks i.e. higher cognitive loads (e.g. encoding and recall of novel faces) are able to reveal differences in brain activation level^76, 77^. Thus, imaging performed under so-called challenging “cognitive stress” tests have a higher potential to reveal subtle alterations in brain function, perhaps prior to the emergence of mild cognitive impairments seen at early stages of AD^78^. Paired associate learning, particularly the pairing of a name to a face, has been suggested to be quite useful in early detection of memory impairment as it targets the most common problem among elderly^16, 17^. Thus, our next step is to perform measurements on healthy elderly and patients with memory deficits to test the cognitive load hypothesis and the potential of fNIRS in unmasking the underlying brain pathology early on in the disease.

## Conclusion

This study demonstrates that fNIRS can robustly measure memory encoding and retrieval-related brain activity, namely oxy and deoxy-hemoglobin concentration changes in prefrontal, temporal and parietal cortices. Our findings are consistent with previous fMRI and PET literature. The brain response during the recalling phase was both high in amplitude and has a broader spatial extent compared to the brain response during the encoding phase, and the hemodynamic response amplitude is found to be correlated with the memory performance. Measurements on healthy elderly and patients with memory deficits such as patients with MCI or AD are needed to further test the ability of fNIRS to differentiate the brain responses of different populations under various cognitive loads. If characteristic changes of hemodynamic response to encoding and retrieval can be linked with MCI, fNIRS may offer a strong biomarker for early detection of memory impairment.

## Methods

### Participants

Nineteen healthy subjects (age: 31±9.7 years, 11 male, 8 female) were recruited for this study. Written informed consent was obtained from all subjects. The study was approved by Massachusetts General Hospital (MGH). The methods were carried out in accordance with the guidelines and regulations of the Institutional Review Board of MGH. Eighteen participants were right-handed and 1 was left handed. Subjects had no neuropsychological disorder and they had normal or corrected to normal vision.

### Probe placement

The NIRS probe was designed utilizing AtlasViewer software^79^. The probe design and the probe itself can be seen in Fig. 9. The probe consisted of 14 sources, 18 long separation detectors and 14 short separation detectors that are located 30 and 8 mm away from the sources respectively. This configuration resulted in a total of 54 channels in which 40 are long and 14 are short separation source-detector pairs. The channels are named from 1 to 54 and only the names of long separation channels are shown in Fig.8 and Fig. 9. The probe covered the frontal cortex along with superior temporal and inferior parietal regions.

### fNIRS system

Data were acquired by using a CW7 NIRS system utilizing laser diodes at 690 and 830nm (TechEn Inc. MA, USA) and acquiring signals from the photo-detectors at an acquisition frequency of 25 Hz. The TechEn system is a multichannel continuous wave optical imager consisted of 32 frequency-encoded lasers (half of them at 690 and the other half at 830 nm) and 32 avalanche photo-diode detectors. The light is carried from the CW system to the head probe via optical fibers and is delivered from the head probe back to the CW system via detector fiber bundles.

### Experimental design

**Figure 10:**
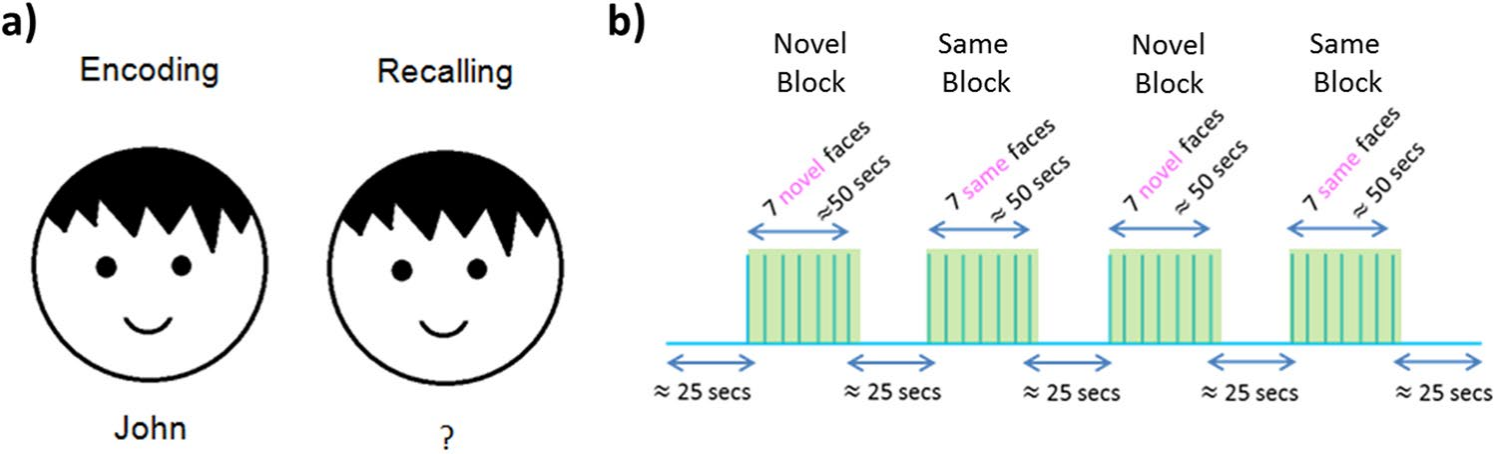
(**a**) Face-name paired-associate paradigm, (**b**) Schematic diagram of the task design for both encoding and recalling runs.

A face-name PAL task was used in this study that has previously been used in an fMRI study^14^. The task requires a set of paired faces and names. For this, we have randomly combined neutral faces obtained from The Face Database^80^ and the most popular given names for male and female during the last 100 years from varying age and ethnicity obtained from USA Social Security Administration. The stimuli were presented using a personal computer. A schematic diagram of the task design is shown in Fig. 10. Each run consisted of two novel face-name pair blocks and two same face-name pair blocks, presented in the order of novel, same, novel, and same. Each block lasted ~50 secs in which 7 face-name pairs were displayed sequentially. Faces were presented on a white background on the screen for 4.5 secs and there was a 3–3.5 sec interval between each face-name pair trial. Each run started with a 25 sec baseline and each block was followed by a ~25 sec reset period. Thus, each run consisted of 4 blocks and lasted for ~325 sec. The experimental design parameters were the same for encoding and recalling runs.

During the rest periods, subjects were shown a black screen. In total, the session was composed of two encoding runs and two retrieval runs, presented in the order of encoding, retrieval, encoding, and retrieval. During the encoding runs, face-name pairs were presented sequentially and subjects were asked to memorize each face-name pair. During the retrieval, each face was shown without the paired name and the subjects were asked to recall and tell the name that matches the face. The retrieval experiment was executed after a delay lasting more than 1 minute and less than 5 minutes after each encoding run. The subject responses were recorded for further analysis.

Prior to the experiment, the procedure was explained to the participants and the same face-name pairs (one male and one female) i.e. the control faces were shown to them in order to make subjects familiar with these pairs. This way, we can avoid inducing any brain activation due to the encoding of the control face-name pairs. These two face-name pairs were shown during the control runs interchangeably. During the experiment, all participants were seated in front of a laptop in a quiet room. The face-name PAL task session lasted 24 min. Following the NIRS data acquisition, for each subject, the 3D positions of the sources and detectors as well as the EEG landmarks (inion, nasion, ears and Cz) were acquired by using a 3D digitizer (Polhemus Inc., VT).

### Data Analysis and Statistics

fNIRS data were processed by using open source software HOMER2 which is implemented in MATLAB (Mathworks, Natick, MA)^81^. First, the raw NIRS signals were converted to optical density and then only the channels lower than 80dB and higher than 140dB were excluded from further analysis. For our fNIRS system, the signal to noise ratio is typically less than 10 when the signal is below 80 dB, and the signal saturates above 140 dB (these thresholds will be different on different systems). For each channel, the total number of subjects left after the data quality check, is presented in Fig. 8, column 2. A low-pass filter with a cut-off frequency of 0.5 Hz was applied to OD signals to remove high-frequency noise. Then, the OD signals were converted to oxygenated-hemoglobin (HbO) and deoxygenated-hemoglobin (HbR) concentrations by using the modified Beer-Lambert law with a partial pathlength factor of 6^82, 83^. The trials that were objectively determined to be affected by motion artifacts were excluded from further analysis^81^. The hemodynamic response function (HRF) was then estimated by using a general linear model (GLM) which uses the least squares method for estimating the weights of consecutive basis functions. The GLM is used to get the time course (rather than a single beta coefficient for a canonical HRF described by a single temporal basis function). Thus, the group average result is of the time courses of the whole HRF for each subject, not of a beta coefficient for a single temporal basis function^84^. The GLM is done with a series of several Gaussian functions with a standard deviation of 0.5 sec and their means separated by 0.5 sec as we have done in prior studies^85, 86^. We modeled the baseline drift with a 3^rd^ order polynomial. To reduce the effect of physiological interferences in the hemodynamic response estimation, the highest correlated short separation (SS) channel with a given long separation (LS) channel is used as a regressor. The principle behind using the short separation regression for canceling the physiological interferences is that the light that passes through superficial layers is collected by the SS channel and the LS channel has the information of functional hemodynamic changes accompanied with hemodynamic information from both superficial layers and the brain. Hence taking the SS channels as regressors to filter systemic interferences from LS channels leads to a more accurate estimation of the brain HRF^86^. Paired student’s t-tests were used to evaluate statistically significant differences in hemodynamic responses to novel and same face stimuli in the time range of 25 to 50 seconds. We used the time range of 25 to 50 seconds because in this time range the hemodynamic signal reaches its steady state. For statistical analysis, multiple comparison correction is applied to each anatomical region (frontal, temporal, parietal cortex) using Benjamini-Hochberg method with a false discovery rate of 0.05^87^. The Pearson coefficient is calculated to get the correlation between accuracy of the recalling of the novel faces and the mean of HbO and HbR change in a time interval of 25 to 50 seconds during each recalling block.

In order to permit comparison with the fMRI literature, the MNI coordinates were estimated using the procedure described by Tsuzuki *et al*.^88^. Briefly, an atlas model of brain and head geometry was scaled to the subject head size using the 3D locations of EEG landmarks obtained by the 3D digitizer. The 3D locations of the optodes were then pulled towards the head surface along the surface normal and then projected to the cortical surface permitting estimation of the corresponding MNI coordinate for each optode as well as the mid-point of each source-detector pair. These processes are done by using the AtlasViewer software^79^.
